# Piezoelectric Energy Harvesting Solutions

**DOI:** 10.3390/s140304755

**Published:** 2014-03-10

**Authors:** Renato Caliò, Udaya Bhaskar Rongala, Domenico Camboni, Mario Milazzo, Cesare Stefanini, Gianluca de Petris, Calogero Maria Oddo

**Affiliations:** 1 The BioRobotics Institute, Scuola Superiore Sant'Anna, Polo Sant'Anna Valdera, Viale Rinaldo Piaggio 34, Pontedera 56025, PISA, Italy; E-Mails: u.rongala@sssup.it (U.B.R.); d.camboni@sssup.it (D.C.); m.milazzo@sssup.it (M.M.); c.stefanini@sssup.it (C.S.); 2 Telecom Italia, WHITE Lab, Via Cardinale Maffi 27, Pisa 56126, PISA, Italy; E-Mail: gianluca.depetris@telecomitalia.it

**Keywords:** energy harvesting, piezoelectric generator, power management, MEMS, wearable technology

## Abstract

This paper reviews the state of the art in piezoelectric energy harvesting. It presents the basics of piezoelectricity and discusses materials choice. The work places emphasis on material operating modes and device configurations, from resonant to non-resonant devices and also to rotational solutions. The reviewed literature is compared based on power density and bandwidth. Lastly, the question of power conversion is addressed by reviewing various circuit solutions.

## 1. Introduction

*Energy harvesting* or *energy scavenging* is the process of extracting small amount of energy from ambient environment through various sources of energy. The available energy for harvesting is mainly provided by ambient light (artificial and natural lighting), ambient radio frequency, thermal sources and mechanical sources.

Reduction in size and energetic demands of sensors, and the low power consumption trend in CMOS electronic circuitry opened novel research lines on battery recharge via available power sources. Harvesters can be employed as battery rechargers in various environments, such as industries, houses [1,2], the military (as for unmanned aerial vehicles [3]) and handheld or wearable devices [4–9]. The possibility to avoid replacing exhausted batteries is highly attractive for wireless networks (Wireless Sensor Networks [10]), in which the maintenance costs due to battery check and replacement are relevant. Another emerging field of application is biomedical systems, where the energy could be harvested from an off-the-shelf piezoelectric unit and used to implement drug delivery systems [11] or tactile sensors [12–14]. Recent research also includes energy conversion from the occlusal contact during chewing by means of a piezoelectric layer [11,15] and from heart beats [16].

**Figure 1.**
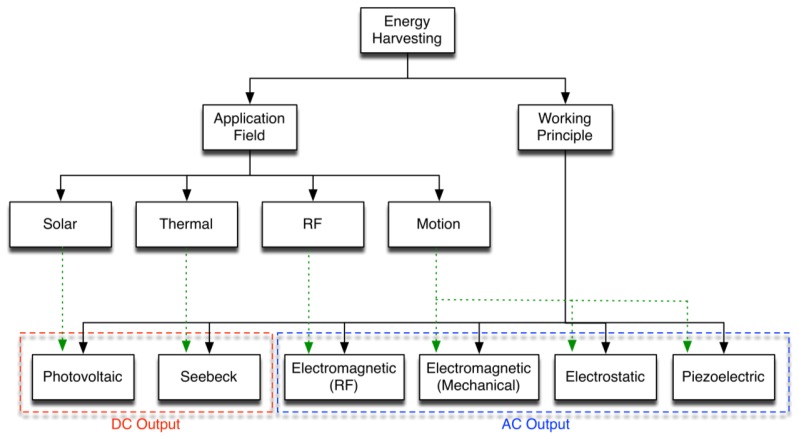
Hierarchy of main energy harvesting technologies.

We can classify the main energy harvesting technologies by the hierarchy shown in Figure 1. Motion harvester systems can be structured as follows: the harvester collects inputs through its frame, directly connected to the hosting structure and to the transducer; at the end of the system chain, a conditioning circuit manipulates the electrical signals. This paper specifically focuses on piezoelectric motion harvesting techniques.

**Figure 2.**
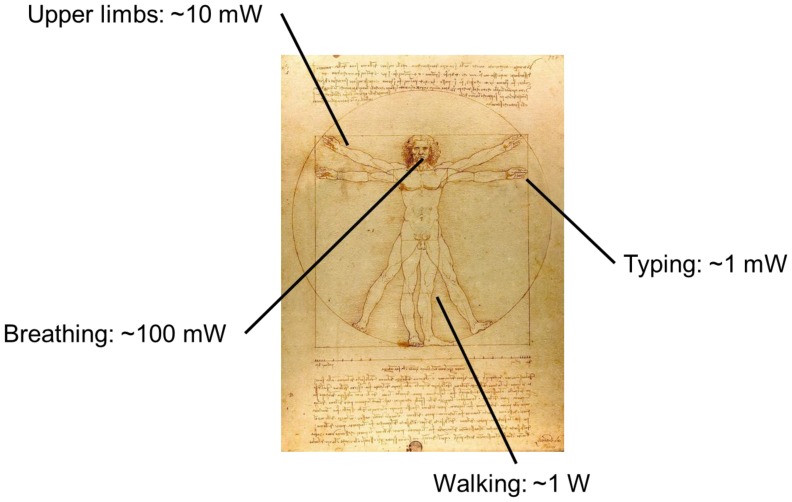
Estimation of available power that could be harvested during human activities (Adapted from [22]).

The possibility and the effectiveness of extracting energy from human activities has been under study for years [17]. As a matter of fact, continuous and uninterrupted power can potentially be available: from typing (∼mW), motion of upper limbs (∼10 mW), air exhalation while breathing (∼100 mW), walking (∼W) [18,19] (Figure 2), and in this work we review state of the art of motion based energy harvesting.

Among available motion based harvesting techniques, piezoelectric transduction offers higher power densities [20] in comparison to electrostatic transduction (which also needs an initial polarization). Also, piezoelectric technologies are better suited than electromagnetic ones for MEMS implementation, because of the limitations in magnets miniaturization with current state-of-the-art microfabrication processes [21].

## 2. Transduction Principle

The piezoelectric effect converts mechanical strain into electric current or voltage. It is based on the fundamental structure of a crystal lattice. Certain crystalline structures have a charge balance with negative and positive polarization, which neutralize along the imaginary polar axis. When this charge balance is perturbed with external stress onto the crystal mesh, the energy is transferred by electric charge carriers creating a current in the crystal. Conversely, with the piezoelectric effect an external charge input will create an unbalance in the neutral charge state causing mechanical stress.

The connection between piezoelectricity and crystal symmetry are closely established. The piezoelectric effect is observed in crystals without center of symmetry, and the relationship can be explained with monocrystal and polycrystalline structures.

**Figure 3.**
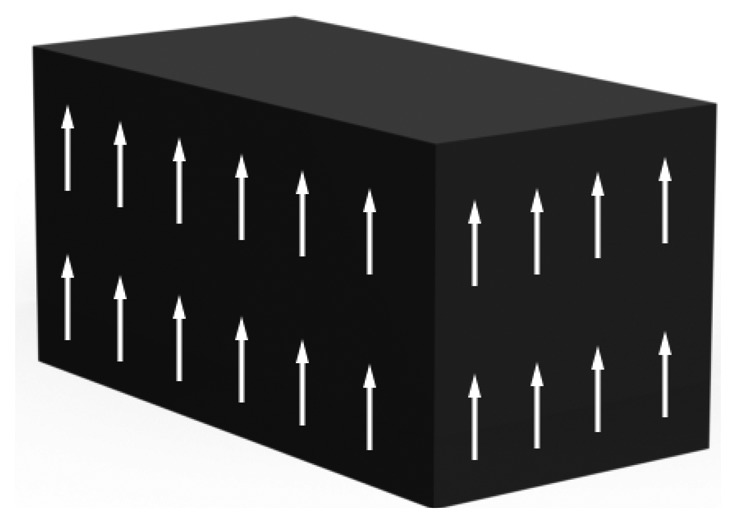
Monocrystal.

In a monocrystal (Figure 3) the polar axes of all of the charge carriers exhibit one-way directional characteristics. These crystals demonstrate symmetry, where the polar axes throughout the crystal would lie unidirectional even if it was split into pieces.

**Figure 4.**
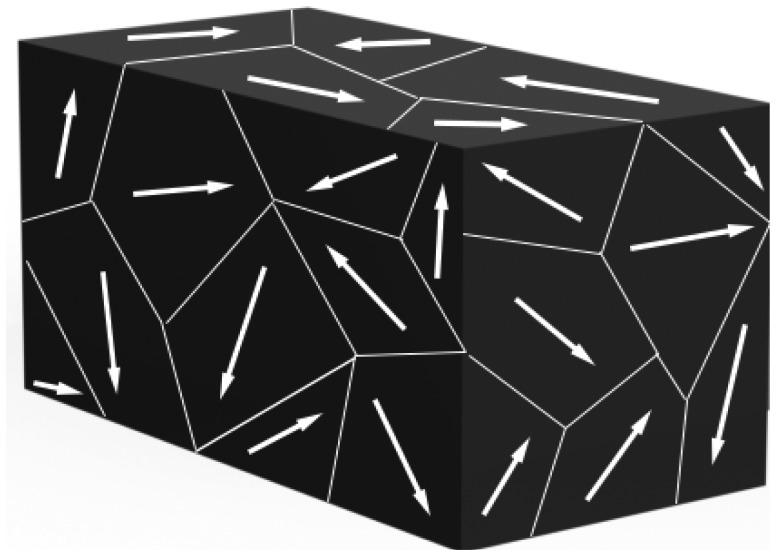
Polycrystal.

Instead, a polycrystal (Figure 4) is characterized by different regions within the material with different polar axes. It is asymmetrical because there is no point at which the crystal could be cut that would leave the two remaining pieces with the same resultant polar axes.

**Figure 5.**
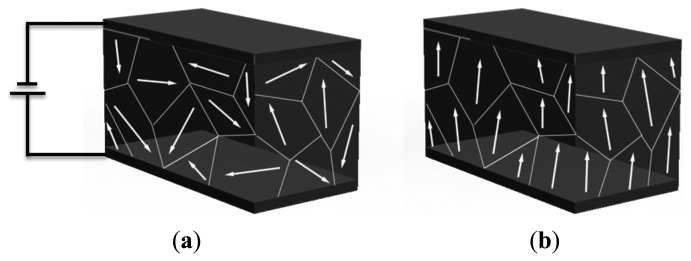
(**a**) Polarizations; (**b**) Surviving Polarity.

In order to attain the piezoelectric effect, the polycrystal is heated to the Curie point along with strong electric field. The heat allows the molecules to move more freely and the electric field forces the dipoles to rearrange in accordance with the external field (Figure 5).

As a result, the material possesses piezoelectric effect: a voltage of the same polarity as of the poling voltage appears between electrodes when the material is compressed; and opposite polarity appears when stretched. Material deformation takes place when a voltage difference is applied, and if an AC signal is applied the material will vibrate at the same frequency as the signal [23–25].

Piezoelectricity is governed by the following constitutive equations, which link the stress *T*, the strain *S*, the electric field *E* and the electrical induction *D*:

(1) $$\{\begin{array}{ll} T_{p} & =c_{pq}^{E}S_{q}-e_{kp}E_{k}\\ D_{i} & =e_{iq}S_{q}+{ɛ}_{ik}^{S}E_{k} \end{array}$$

where $c_{pq}^{E}$ is the Young's modulus, *e_kp_* is the piezoelectric coefficient and ${ɛ}_{ik}^{S}$ is the clamped permittivity. The same relationship can be written in other three forms, depending on the couple of variable (among *T*, *S*, *E* and *D*) chosen to be independent [26]. The superscript *E* indicates a constant electric field (which corresponds for example to a short circuit condition, where *E*=0), as well as the superscript *S* stands for a condition of constant strain.

For each couple of constitutive equations there is a different piezoelectric coefficient, defined as:

(2) $$\begin{array}{c} e_{ip}≜{\left(\frac{\delta D_{i}}{\delta S_{p}}\right)}^{E}=-{\left(\frac{\delta T_{p}}{\delta E_{i}}\right)}^{S}\\ d_{ip}≜{\left(\frac{\delta D_{i}}{\delta T_{p}}\right)}^{E}={\left(\frac{\delta S_{p}}{\delta E_{i}}\right)}^{T}\\ g_{ip}≜-{\left(\frac{\delta E_{i}}{\delta T_{p}}\right)}^{D}={\left(\frac{\delta S_{p}}{\delta D_{i}}\right)}^{T}\\ h_{ip}≜-{\left(\frac{\delta E_{i}}{\delta S_{p}}\right)}^{D}=-{\left(\frac{\delta T_{p}}{\delta D_{i}}\right)}^{S} \end{array}$$

Related to each other as follows:

(3) $$\begin{array}{c} d_{ip}={ɛ}_{ik}^{T}g_{kp}\\ e_{ip}=d_{iq}c_{qp}^{E}\\ g_{ip}=d_{kp}/{ɛ}_{ik}^{T}\\ h_{ip}=g_{iq}c_{qp}^{D} \end{array}$$

An important parameter is the electromechanical coupling factor, *k_iq_*, which describes the conversion between mechanical and electrical energy. It can be written in terms of coefficients of the material:

(4) $$k_{iq}^{2}≜\frac{W_{i}^{\left(\text{electrical}\right)}}{W_{q}^{\left(\text{mechanical}\right)}}=\frac{e_{iq}^{2}}{{ɛ}_{ik}^{T}c_{pq}^{E}}=\frac{e_{iq}^{2}}{{ɛ}_{ik}^{S}c_{pq}^{E}+e_{iq}^{2}}$$

The efficiency of energy conversion, *η*, is described, at resonance, as follows:

(5) $$\eta =\frac{\frac{k^{2}}{2(1-k^{2})}}{\frac{1}{Q}+\frac{k^{2}}{2(1-k^{2})}}$$

where, *k*^2^ is the coupling factor as defined in Equation (4) and Q is the quality factor of the generator [21,27].

To understand how the electrical quantities (*V* and *I*) are related to the mechanical ones (force *F* and displacement *z*), the particular case of a piezoelectric disk can be considered. In this case, from Equation (1) the following relationships can be obtained [28]:

(6) $$\{\begin{array}{l} F_{P}=k_{PE}z+\alpha V\\ I_{p}=\alpha \dot{z}-C_{p}\frac{dV}{dt} \end{array}$$

In which the featuring quantities are the restoring force *F_p_* of the piezoelectric material, its stiffness when it is short-circuited *k_PE_*, the displacement *z*, the force factor *α*, the voltage across the electrodes *V* and the outgoing current *I_p_*, and the clamped capacitance *C_p_*. These equations are derived considering the following approximations:

(7) $$E=-\frac{V}{H};S=\frac{z}{H};I_{p}=A\frac{dD}{dt};F_{P}=AT_{p}$$

and the featured quantities can be written as:

(8) $$k_{PE}=\frac{c_{pq}^{E}A}{H};C_{p}=\frac{{ɛ}_{ik}^{S}A}{H};\alpha =\frac{e_{iq}A}{H}$$

where, *A* and *H* are the section and thickness of the piezoelectric disk.

In a more generic case of a mechanical stress in direction *p* and an induced electric field in direction *i*, the open-circuit voltage of a piezoelectric device can be written as follows:

(9) $$V=T_{p}g_{ip}\text{l}$$

Assuming that the voltage coefficient *g_ip_* is constant with the stress, and where *l* is the gap between the electrodes.

## 3. Materials

Each piezoelectric material can be characterized with a set of parameters. For example, considering a stress *T_p_* as input, the strain coefficient *d_ip_* gives the relationship between the applied stress and the electric induction *D_i_* (therefore, current density is $J_{p}=\frac{dD}{dt}$), while the voltage coefficient *g_ip_* gives the voltage Equation (9). Thus, a high energy density piezoelectric material is characterized by a large product of the strain coefficient (*d_ip_*) and the voltage constant (*g_ip_*) [29]. The coupling factor *k_iq_*, combining the piezoelectric properties of the material with its mechanical and electrical properties, gives the converted energy and efficiency of the harvester, as remarked by Equations (4) and (5). The mechanical characteristics (Young's modulus) define the robustness and toughness of the device and also play an important role in defining piezoelectric coefficients, Equation (3), and coupling factor. Dielectric permittivity also plays a similar role in definitions. All these parameters (determined by the material) are crucial in designing a harvesting system, making in turn material selection a primary factor in piezoelectric harvesters.

Table 1 lists some of the most common piezoelectric materials, mainly piezoceramics (that are polarized ferroelectric ceramics [26]), such as PZT [30] and barium titanate [31]. Out of them, Anton and Sodano [32] and Shen and colleagues [33] report PVDF polymer and micro-fiber composites (MFC) as highly flexible materials. MFCs are composites that combine the energy density of piezoceramic materials with the flexibility of epoxy [34]. In [33], the authors compared PZT with PVDF and MFC, they showed that although PZT shows the highest power density, it is not well suited for high g-vibrations because of its lower yield strength that results in lower robustness, leading to fracture. Furthermore, zinc-oxide (ZnO) is an interesting material that is pushing the piezoelectric field to a nanometric scale. It is used to grow one dimensional hair-like nanowires, with diameters in the sub-one hundred nanometer scale and lengths ranging from several hundreds of nanometers to a few centimeters. Zinc exhibits both semiconductor and piezoelectric properties, it is relatively biosafe and biocompatible, so it can be involved in biomedical applications with little toxicity [35]. In [36] a strain coefficient of ∼10 pC/N was reported for zinc oxide nanowires.

The piezoelectric properties change logarithmically with age allowing them to stabilize. Therefore, manufacturers usually specify the constants of the material after a period of time [21,37]. Table 1 compares a set of piezoelectric materials based on the coefficients related to mechanical input and electrical output.

## 4. Resonant Devices

Piezoelectric transducers are frequently used in inertial generators. These systems are composed of a fixed reference, which transmits vibrations to an inertial mass located on a mechanical moving part, when an external acceleration is applied. Piezoelectric materials provide transduction, exploiting the mechanical strain occurring in such devices.

**Figure 6.**
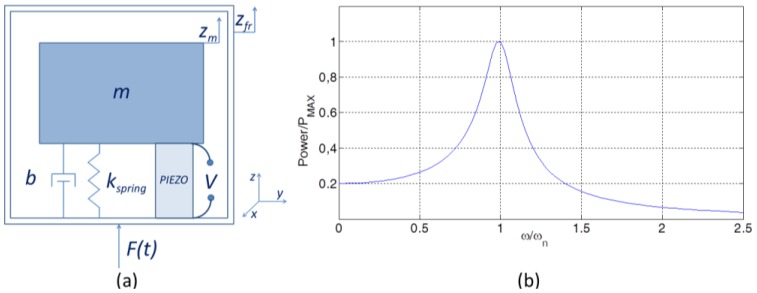
(**a**) Mass-spring-damper-piezo model and (**b**) its resonant behavior.

Inertial generators can be described as a second order mass-spring-damper system [29,43–45], along with a piezoelectric element connected parallel to the damper [28] (Figure 6). The system is governed by the following equation of motion:

(10) $$m{\ddot{z}}_{m}(t)+b{\dot{z}}_{m}(t)+kz_{m}(t)=F(t)$$

where *m*, *b* and *k* are seismic mass, total damping coefficient and spring stiffness respectively, while *F(t)* is an external force applied onto the device. The damping coefficient *b* comprises of both mechanical losses and coefficient based on energy conversion, which are *b_p_* and *b_e_*, respectively. The total stiffness of spring accounts to the spring stiffness (*k_spring_*) and the piezoelectric stiffness (*k_piezo_*). This is a good approximation as long as the structure vibrates with little displacements and the mechanical behavior of motion remains linear.

Such a system is characterized by a natural or resonant angular frequency (*ω_n_*) given by:

(11) $$\omega_{n}=\sqrt{\frac{k}{m}}$$

In practical cases *ω* has to be designed to match the expected ambient excitation angular frequency (*ω*). When this happens, the maximum energy is extracted from the transducer (as shown in Figure 6).

**Figure 7.**
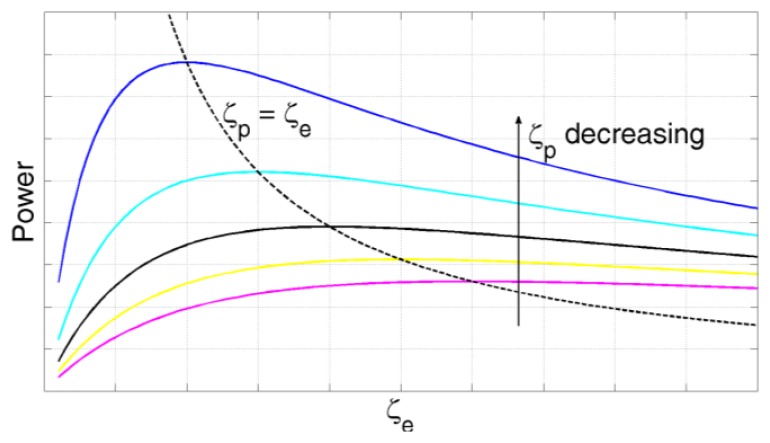
Power of the piezoelectric generator as a function of the electrical damping ratio. The black dashed line is obtained from Equation (12) by imposing *ζ_p_* = *ζ_e_*. For a given mechanical damping, the maximum extracted power is achieved when the electrical damping equals the mechanical damping.

The power that the transducer can extract while working at resonance can be derived solving (8) for *z_m_*. The harvested power is obtained by formulating the portion of the power flowing through the damper related to the transducing mechanism [21,45]:

(12) $$P_{e}=\frac{m\zeta_{e}a^{2}}{4\omega_{n}{\left(\xi_{p}+\xi_{e}\right)}^{2}}$$

where, $a=\omega_{n}^{2}Z$ is the acceleration in the case of sinusoidal vibratory excitation. The displacement of the device is *z_fr_*(*t*)=*Zsin*(*ωt*). The mass is *m*; *ζ_e_* and *ζ_p_* are the transducer and the mechanical damping ratio, respectively (*ζ*=*b*/2*mω_n_*). A high mechanical damping would flatten out the power curve *P_e_*. Power output reaches its maximum when the electrical damping is equal to the mechanical damping [20,46] (Figure 7). Resonant devices are discussed below, based on operating modes (*d*_31_, *d*_33_, *d*_15_).

### 4.1. d_31_ Mode Generators

**Figure 8.**
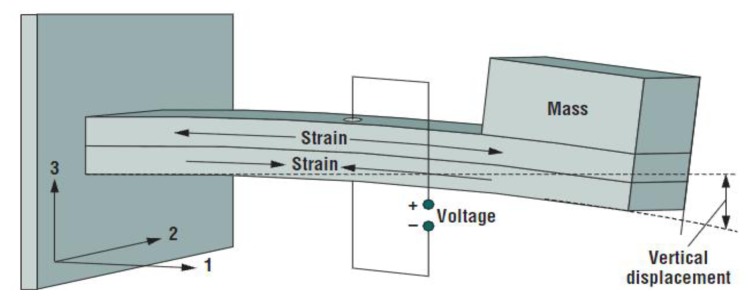
Rectangular cantilever beam (^©^ 2005 IEEE. Reprinted with permission from [47]).

In *d_31_* operating mode the material has an induced electric field in direction 3, as a response to the stress along direction 1. Figure 8 shows the most common configuration of a piezoelectric harvester, which comprises of a rectangular beam, a tip mass and a piezoelectric material.

We can distinguish unimorph and bimorph configurations based on the piezoelectric material presence on top of the beam or on both sides. A thorough comparison of these two configurations was presented by Ng in [48]. The former was described as most suitable for lower frequencies and load resistance, whereas the latter showed optimal functioning at higher frequencies and higher loads, while being able to extract higher amount of power. Indeed, in bimorph cantilever the two piezoelectric pieces undergo opposite strain during operation, and they can be bonded electrically in series (to increase the voltage output) or in parallel (to increase the current output). Lu and colleagues [49] investigated the load resistance effort in case of bimorph cantilevers and pure resistive load. They formulated the following relationship for the optimal load:

(13) $$R_{\text{opt}}=\frac{t}{WL{ɛ}_{33}\omega }=\frac{1}{C_{p}\omega }$$

where, *t* is the thickness, *L* the length of the piezoelectric layer, *W* the beam width, *ε_33_* the dielectric constant, *ω* the angular frequency and *C_p_* the parasitic capacitance of the piezoelectric material. This shows that the optimal resistance varies with geometrical configurations and material properties.

**Figure 9.**
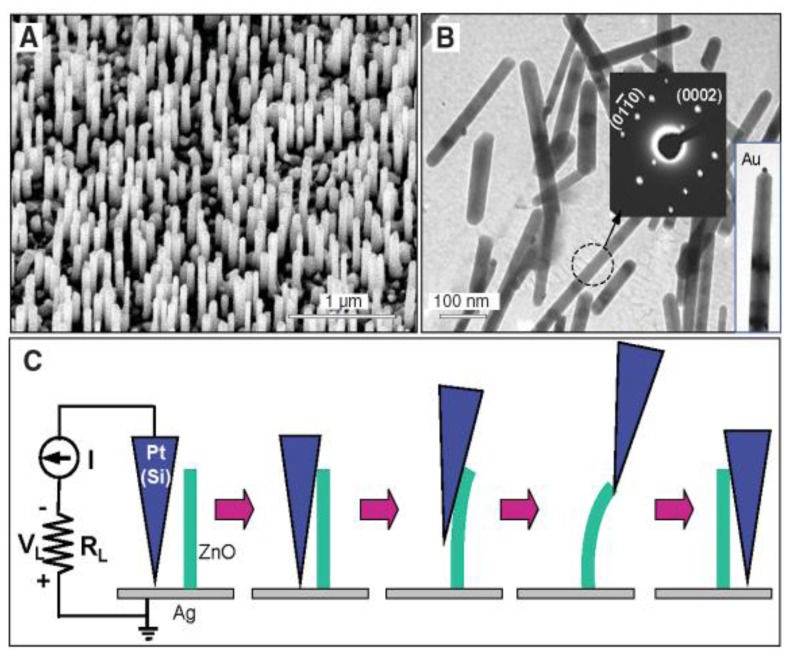
Nanowire array based generator presented by Wang and Song from [35] (Reprinted with permission from AAAS).

Wang and Song [35] developed a nanogenerator based on a zinc oxide nanowire array (Figure 9A). A silver paste was used as ground contact at the bottom of the nanowires, while the other electrodes were fabricated by means of a Pt film coated on the tip of an atomic force microscope (Figure 9C). Pt was chosen in order to form a Schottky barrier at the interface with ZnO. The barrier allows the nanowire to accumulate charge during the deformation along its length (due to the piezoelectric effect) and discharging it on a load circuit only when the Schottky barrier is forward biased. This happens only during a small portion of the entire cycle, but resulting in a sharper and higher voltage (and power) output. The authors reported conversion efficiency ranging between 17% and 30%, and a typical resonance frequency of ∼10 MHz. Each nanowire is estimated to provide with an output voltage of ∼8 mV and corresponding power of ∼0.5 pW. Considering an array of 20 nanowires per μm**^2^**, the output power can be ∼10 pW/μm**^2^**.

**Figure 10.**
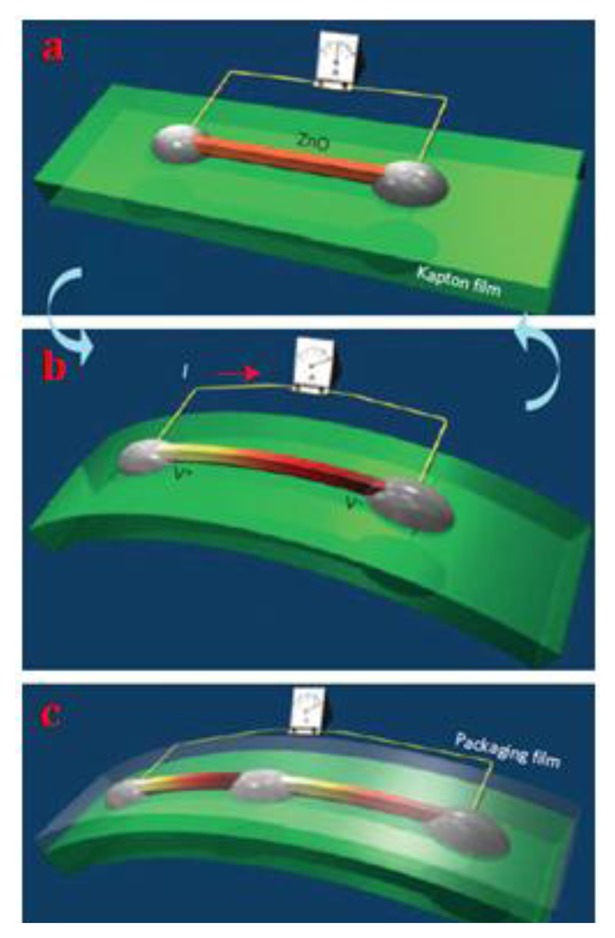
Design of a piezoelectric fine wire (PFW) generator on a flexible substrate [50] (Reprinted with permission from Macmillan Publishers Ltd.).

Vertically aligned piezoelectric nanowires may lack in mechanical robustness, lifetime, environmental adaptability and output stability. To overcome such inconveniences, a flexible power generator based on cyclic stretching-releasing mechanism for piezoelectric fine wires was proposed by Yang and colleagues [50]. In this work, a piezoelectric fine wire (PFW) lies flat on a flexible substrate with fixed ends onto the electrodes (Figure 10a). When the substrate is subjected to load, it bends (Figure 10b) inducing a tensile strain of 0.05%–0.1% in the wire. This leads to a drop in the piezoelectric potential along the wire, forcing electrons to flow along an external circuit to charge the wire. When the substrate is released, electrons flow back in the opposite direction. Periodically bending and releasing the PFW therefore generates an alternating current. Generators based on multiple PFWs can be integrated to raise the output voltage.

### 4.2. d_33_ Mode Generators

In *d*_33_ operating mode the material is subjected to a stress in the same direction of the produced electric field. This operating mode led initially to impact harvesters [51–55], while vibrating generators were made only via *d*_31_ effect. However, several researchers focused on fabricating vibrating devices with alternative modes to *d*_31_. This research line is motivated by the fact that piezoelectric coefficients in *d*_33_ and *d*_15_ modes are higher than *d*_31_ ones (Table 1), so this can possibly lead to devices with higher output power. *d*_33_ vibrating generators can be used, for example, in industrial fields such as automotive and machinery or wherever there are mechanical joints that, due to tolerances, show relative movements between structural components. The cyclical movement of structures, due for example to the effect induced by the vibrational dynamics of the system, can be exploited by adopting appropriate mechatronic systems. Thanks to their geometry and to their structure, the mechanism is able to convert macroscopic displacement (in the order of millimeters) into a high force microscopic motion acceptable by the piezoelectric material.

**Figure 11.**
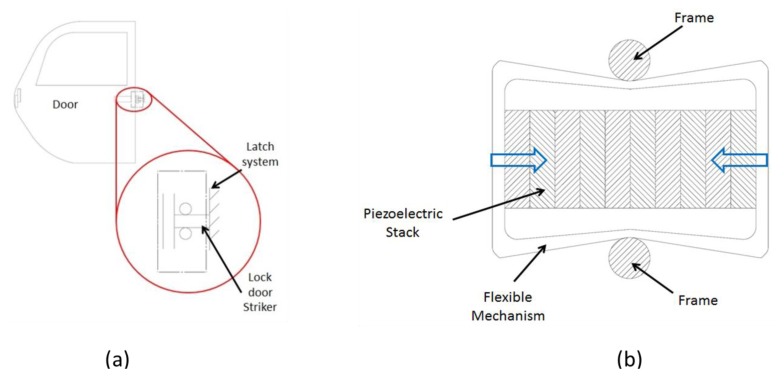
(**a**) Car door latch system; (**b**) a possible architecture of the harvester.

As an example, a possible application of *d*_33_ generators is represented by the car door latch system shown in Figure 11a. This assembly shows a cyclic displacement (highest value ∼1 mm) with frequency range between 0 and 10 Hz. In this view, it is possible to design an electromechanical *d*_33_ system with a metal frame, to be coupled with the internal part of the closure, able to scale the displacement in values compatible with piezostacks (Figure 11b).

**Figure 12.**
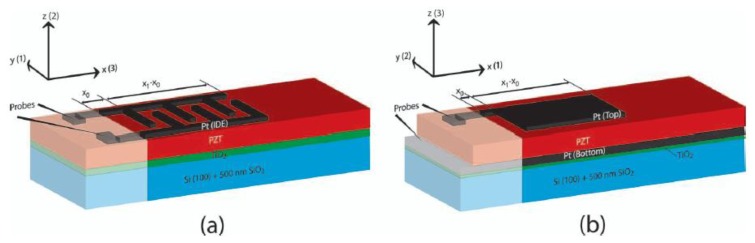
Comparison between (**a**) Interdigitated electrode (IDE) and (**b**) Top and bottom electrode (TBE) configurations (^©^ 2012 IEEE. Reprinted with permission from [56]).

**Figure 13.**
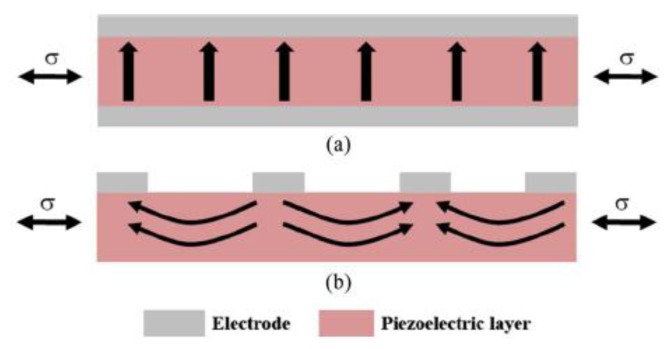
Polarization with d_31_ mode (**a**) and d_33_ mode (**b**) piezoelectric harvesters (^©^ 2013 IEEE. Reprinted with permission from [57]).

Several *d*_33_ operating modes were implemented with vibrating harvesters by using an interdigitated electrode patterning (IDE) instead of the top and bottom electrode (TBE) or parallel plate electrode (PPE) configurations of the *d*_31_ devices (Figure 12). In this way the *d*_33_ mode is obtained by letting the direction-3 to coincide to the length direction of the beam (Figure 13).

Typically a *d*_33_ mode harvester develops a greater output voltage than *d*_31_ devices because of its greater voltage coefficient (*g*_33_ > *g*_31_), and large gap between the electrodes. In *d*_33_ generators the limiting factor is the length of the piezoelectric material instead of its thickness, hence the gap between electrodes can be made larger than that of *d*_31_ devices. However, it is important to notice that greater electrode spacing requires higher voltage in order to polarize the piezoelectric material.

Furthermore, as it can be seen from Figure 13, the interdigitated configuration does not permit the efficient polarization of the material, resulting in curved polarization arrows. Therefore, non-polarization occurs along direction-3, whereas polarization follows in direction-1 just below the electrodes. This type of inefficiency leads to poor performance, in terms of output power.

Several research papers were published, attempting to analyze and unravel the problem of inefficient polarization [58–60]. In particular, Knight and colleagues [60] came up with finite element analysis resulting in a 0.8 optimal ratio between the width of each electrode fingers and the thickness of piezoelectric material, while the ratio between width of the cantilever beam and the finger spacing should be as large as possible. *d*_33_ and *d*_31_ unimorph configurations were also compared in terms of charge generated, by introducing a parameter that accounts for the percentage of the 3-direction polarized material (%*d*_33_). They concluded that, since *d*_33_ coefficient is roughly two times *d*_31_, (%*d*_33_ should be at least 50%, otherwise the IDE configuration has lower efficiency than the TBE one.

To date several IDE devices have been presented [57,61–66]. Jeon and colleagues developed an IDE micropower generator [61,62]. It employed an interdigitated Ti/Pt electrode patterned on top of PZT, its dimensions were submillimetric and it was able to harvest about 1μW power (2.4 V_dc_ after a rectifying stage) at a resonance frequency of 13.9 kHz. Furthermore, a new topology of serpentine cantilever [62] was proposed in order to increase the length of the device maintaining a limited size, but no prototypes were fabricated.

In 2010, Park and colleagues [63] fabricated and compared two millimetric piezoelectric harvesters involving *d*_33_ and a *d*_31_ modes, with same dimensions and resonant frequency. Later, the same group compared two submillimetric *d*_33_ and a *d*_31_ harvesters [57,64], after an experimental optimization of the *d*_33_ device by fabricating a set of twelve different versions of the *d*_33_ cantilever with different finger width and finger spacing. In both works higher output power was obtained with the *d*_31_ harvester and higher output voltage with the *d*_33_ device [57,63,64].

An innovative energy harvester was presented by Zhou and colleagues based on self-biased magneto-electric response [67,68], combining magnetic and piezoelectric vibrational energy harvesting. The device consists of a piezoelectric Macro-Fiber Composite (MFC, M-4010-P1, Smart Material Corp., Sarasota, FL, USA) bonded onto a magnetostrictive Ni cantilever. This device has three different operating modes (see Figure 1 for classification), mechanical electromagnetic, piezoelectric and hybrid respectively. In pure piezoelectric mode, the device harvests 168 μW on a 4 MΩ load, for a mechanical vibration of 0.17 g at 22.5 Hz frequency.

Delnavaz and Voix [69] studied the possibility of harvesting energy from ear canal dynamic motion and designed two micro-power generators, one of which is piezoelectric based. It consists of a flexible sheet of PVDF (provided by Measurement Specialties, [70]), so that the device is biocompatible [69,71]. The device is fabricated by cutting a T-shape from the piezo sheet, joining the tips of the T cross and letting the stem of the T as a tail, thus forming a ring-like device. This structure is mounted in a headset and has to be placed inside the ear canal. During mouth movements the piezo-ring will be deformed, therefore generating an electrical output.

In 2010, Qi and colleagues [72] proposed an innovative method to integrate a high efficiency piezoelectric material onto flexible materials. The method consists of printing PZT ribbons with a thickness of few hundreds nanometers, onto a PDMS substrate. It allowed achieving high piezoelectric coefficients with the advantage of flexibility. The same group also presented a flexible and stretchable device from buckled PZT ribbons, showing the possibility to integrate the high performance *d*_33_ PZT with a stretchable device [73].

More recently, Dagdeviren and colleagues [16] employed PZT ribbons in a system able to scavenge energy from movements of heart, lung and diaphragm. The harvester was arranged onto a flexible membrane, which integrates also a rechargeable battery and a bridge rectifier. The whole system was encapsulated with biocompatible layers (polyimide) and the absence of toxicity was examined with rat smooth muscle cells. The system power generation capability was *in vivo* tested on bovine and ovine hearts. Results showed that a stack of five PZT sheets was able to harvest up to 1.2 μW/cm^2^, sufficient to operate a cardiac pacemaker. Such a system can avoid risks of surgical procedures to replace the depleted batteries of implantable devices, as pacemaker, neural stimulators, cardioverter defribillators and so on.

### 4.3. d_15_ Mode Generators

**Figure 14.**
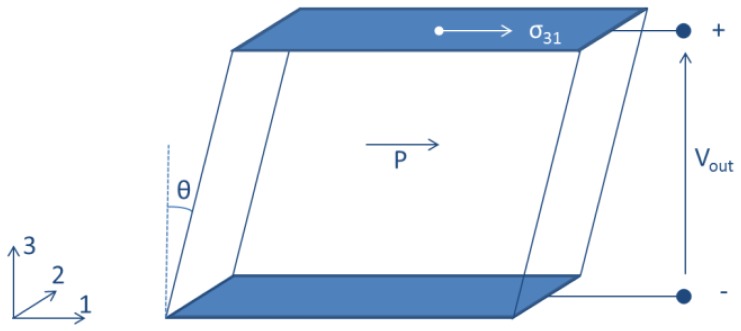
Shear stress harvester (*d*_15_ operating mode).

*d*_15_ operating mode characterizes shear stress harvesters. In this mode the piezoelectric material is polarized along direction 1 and is subjected to a shear stress *σ*_31_. The electrical output is perpendicular to both the polarization and the applied stress. The main problem of these devices is related to the perpendicularity of the polarization direction and the electrical output (Figure 14), which constrains to use a set of electrodes for the polarization and a different set of electrodes for operation.

**Figure 15.**
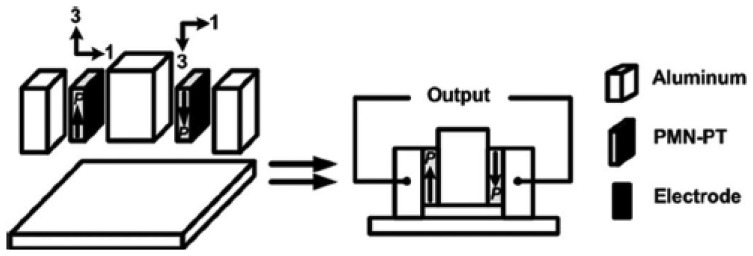
Nonresonant *d*_15_ device presented by Ren and colleagues (^©^ 2010 IEEE. Reprinted with permission from [74]).

Ren and colleagues [74] proposed a non-resonant shear stress harvester involving PMN-PT crystal (last row of Table 1). It comprises two piezoelectric wafers polarized along their length, sandwiched between three aluminum blocks. One block is left free to vibrate with external oscillations (Figure 15). The two piezoelectric wafers were connected in series, with different mass load bonded to the central aluminum block. Under a brass load mass of 200 g and a corresponding acceleration of 121.6 m/s**^2^** at 500 Hz frequency, the device delivered 11.3 V and 0.7 mW on a resistive load of 91 kΩ.

**Figure 16.**
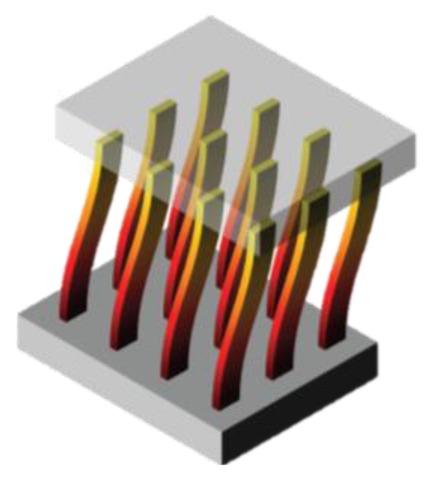
ZnO nanoribbons array presented by Majidi *et al.* [75] (^©^ IOP Publishing. Reproduced with permission of IOP Publishing).

Majidi and colleagues [75] designed a shear-mode ZnO harvester that does not need a nanostructured cathode and allows a permanent bonding of electrodes to the ribbons (Figure 16). The nanoribbons are polarized along their thickness, so that a sliding movement of the electrodes induces voltage drop along the length of nanoribbons. The model developed by the authors predicted that such a system could harvest up to 100 nW/mm^3^.

Wang and Liu [76] developed a piezoelectric energy harvester for pressurized water flow. They employed a PZT-5H in *d*_15_ mode onto a nickel flexible diaphragm that vibrates when the pressure of flow changes. Their device achieved 0.45 nW on a matched load of 1.6 MΩ for a pressure amplitude of 20.8 kPa at 45 Hz frequency.

**Figure 17.**
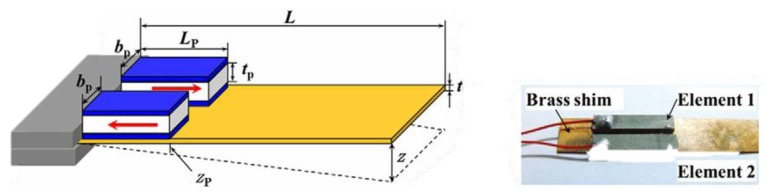
Schematic representation and picture of the *d*_15_ series connected harvester of Zhao and colleagues [77] (**^©^** IOP Publishing. Reproduced by permission of IOP Publishing).

Zhao and colleagues [77] fabricated a piezoelectric harvester with two *d*_15_ PZT-51 connected in series, as shown in Figure 17. They also compared its performances with those of a *d*_15_ single element and demonstrated the higher performances with the series topology.

### 4.4. Comparison among Modes

Resonant piezoelectric generators are the easiest solution for motion harvesting [21]. They can be shaped in simple geometrical configurations by involving a *d*_31_ piezoelectric element. However, different sophisticated solutions were developed in order to increase the extracted power (*d*_15_ devices) or output voltage (*d*_33_ devices) and to scale the technology [35,50,75].

Table 2 summarizes the reviewed literature, by collectively comparing significant parameters for each device to evaluate the power factor, that is defined as the output power normalized with respect to the device volume and input acceleration (*g*).

Based on the analysis of various configurations in the reviewed literature, we can point out that *d*_31_ represents the most used operating mode for piezoelectric-based devices. *d*_33_ vibratory scavengers were fabricated in the attempt to overcome *d*_31_ constraints with power performance. Despite the expectations *d*_33_ devices do not show effective performances, because of polarization issues (Figure 13) due to a percentage of piezoelectric material that does not contribute to energy transduction. However, design optimizations were proposed through finite element simulations [60], but to our knowledge no device was fabricated following such guidelines. As reported in Table 2, *d*_33_ harvesters offer higher voltage output in comparison to *d*_31_ devices. On the other hand, *d*_15_ mode appears to show the best power performances but it requires a complex fabrication process.

From the perspective of employed materials, PZT has been widely used. However, PMN-PT shows higher piezoelectric coefficients with respect to PZT, while ZnO is allowing a rapid growth in nano-scale harvesting technologies. Furthermore, recent studies report high power densities (2.7 mWcm^−3^ in [79]) for ZnO nanowire arrays, that are being used for novel applications such as the detection of facial wrinkling with help of ZnO nanowire-based super-flexible nanogenerator harvesters [80].

## 5. Optimal Shapes

**Figure 18.**
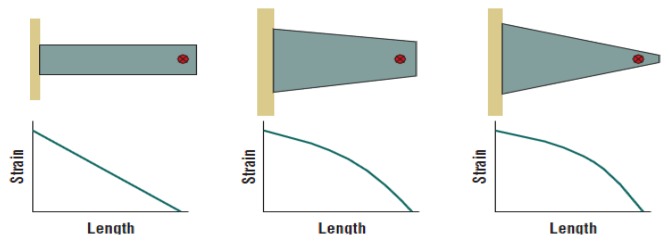
Trapezoidal beams (^©^ 2005 IEEE. Reprinted with permission from [47]).

Rectangular cantilever beams often suffer with over strain at grounded point (near clamping) of the oscillator. Therefore, alternative mechanical structures were investigated in order to prevent overstrain [7,47,81,82]. This goal was achieved by fabricating new possible shapes for cantilever beams, such as trapezoidal beam (Figure 18). These beams distribute the strain more evenly along the structure; they also allow loading the device with higher excitation, leading to duplicate the harvested energy density. Mateu and Moll [7] investigated triangular shaped cantilevers.

Marzencki and colleagues [78,83] developed a MEMS cantilever within the European VIBration Energy Scavenging (VIBES) project, using a deep reactive ion etching on a SOI wafer. They studied the possibility of introducing two angles of curvature at the beginning and at the end of the beam (before the mass) in order to reduce the overstrain [78,83].

## 6. Frequency Tuning

**Figure 19.**
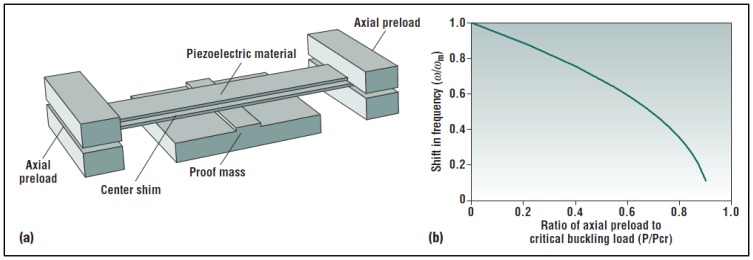
Cantilever with axial load. The right inset shows how the resonating frequency decreases as a function of axial loads. Moreover, the relationship is almost linear up to half the buckling load (^©^ 2005 IEEE. Reprinted with permission from [47]).

Resonant devices have limitations, as described above, because of their narrow bandwidth (approximately equal to 2*ζω_n_*, [47], which typically means few hertz), so they can work efficiently only at their natural frequency (defined by geometry and materials). In this view, several solutions were explored in order to enlarge the bandwidth of the transducer [47,84–86]. Indeed this kind of systems can be used in many applications in which frequency range is wide, due to the environmental conditions. For example in the machinery field, structures are subjected to random, broad spectrum oscillations depending on their specific working principle but also on the wear of the surfaces cyclically in contact. Some passive tuning solutions, such as a moveable clamp that changes the beam length, or nonlinear, bistable structures with destabilizing axial loads are shown in Figure 19.

**Figure 20.**
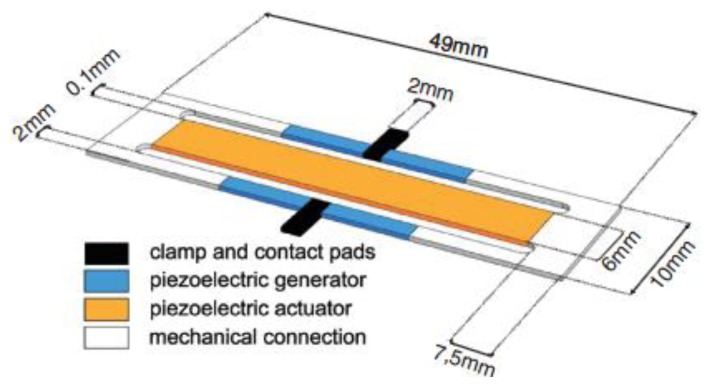
Self-tuning double cantilevered harvester [88] (**^©^** IOP Publishing. Reproduced by permission of IOP Publishing).

Active tuning harvesters were discussed in [87–91]. Eichhorn and colleagues [88] developed an actuated harvester using PZT both for harvesting and actuation. It consists of a two beams device with three arms (Figure 20).

In such a device, the central and larger arm houses the actuator, whilst two harvesters are located in the lateral tight arms. The actuator was designed longer than the transducers, based on previous experimental findings [92]. The control unit mainly consists of a microcontroller, an acceleration sensor and a step-up converter for actuator voltage conditioning. The control unit is equipped with a look-up table in order to avoid a higher power dissipation due to a continued use of the embedded accelerometer. The accelerometer is OFF for most of the time and is turned ON periodically to give a feedback and in case update the look-up table (to overcome aging effects and temperature dependence). The authors reported that this device is capable to harvest up to 90 μW at an acceleration amplitude of 0.6 g. The natural frequency can be tuned within 150 and 215 Hz with actuator voltages between −30 and +45 V. Furthermore, the average consumption of the whole control unit is around 11 μW if the system maintains a constant resonance frequency, however the power consumption can increase by several orders of magnitude if the ambient vibration shifts. Furthermore, it is important to note that the equipped dc-dc converter does not allow negative voltages, so the authors are working on an enhanced device with separated ground electrodes in order to overcome this issue.

Another approach is to enlarge the bandwidth by fabricating an array of cantilevers with different natural frequencies [43,93]. Such device allows to convert energy from different sources at different frequencies, however it reduces the harvested power per unit area because only one cantilever of the array works efficiently for a given source (at a given frequency), while the others do not.

**Figure 21.**
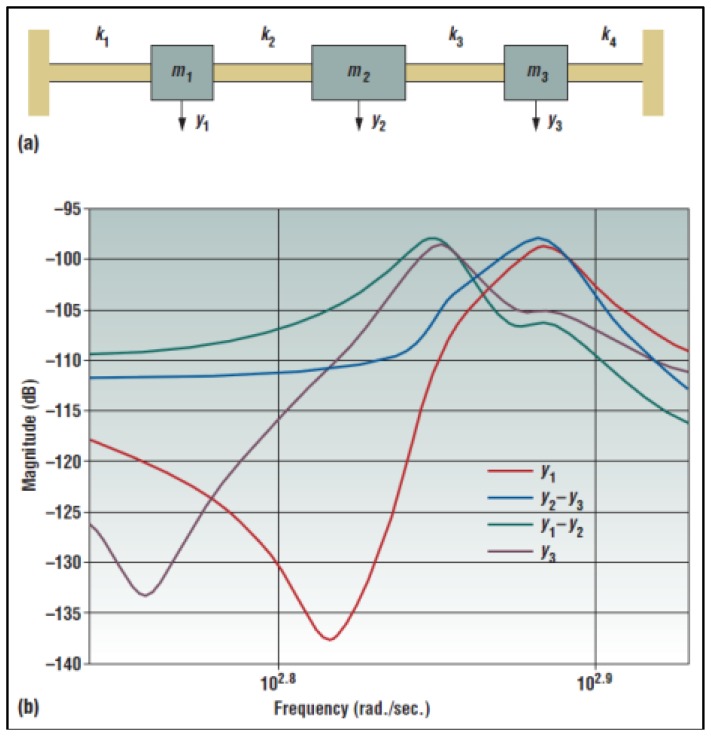
Multimass harvesting system (^©^ 2005 IEEE. Reprinted with permission from [47]).

Roundy and colleagues proposed a similar device obtained connecting three spring-mass-damper systems with different natural frequencies [47]. The resulting device output is almost an overlap of the single subsystems response (Figure 21).

## 7. Non-Resonant Devices

A novel approach based on nonlinear oscillators was proposed by Cottone and colleagues [94]. The authors demonstrated that a bistable oscillator could widen the bandwidth of a traditional resonant system. The design of this device derives from the inverted pendulum; in which reaction forces from two permanent magnets are used (one on the tip mass of cantilever, and the other just in front on an adjustable stage) to provide two stable states.

**Figure 22.**
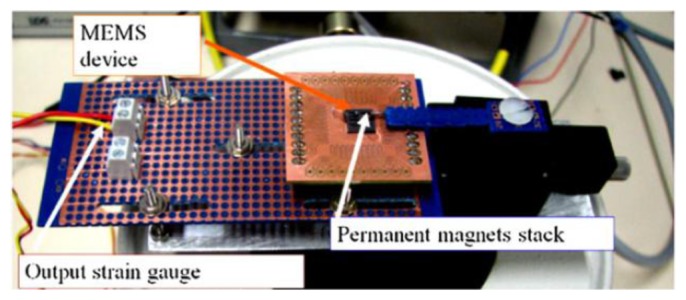
MEMS nonlinear oscillator [96] (**^©^** IOP Publishing. Reproduced by permission of IOP Publishing).

Among all the proposed devices [95–100], Andò and colleagues [96] developed a MEMS harvester based on the bistable oscillator model (Figure 22).

It can be referred to a mass-spring-damper system, similar to Equation (8), but with an additional nonlinear term described by a nonlinear potential energy function:

(14) $$m\ddot{z}+b\dot{z}+\Psi =f(t)$$

where *m* is the mass of beam, *b* is the damping coefficient, *f*(*t*) is the excitation force and Ψ is a nonlinear term (which also includes the elastic constant *k*) [101]:

(15) $$\Psi ≜\frac{\partial U(z)}{\partial z}$$

**Figure 23.**
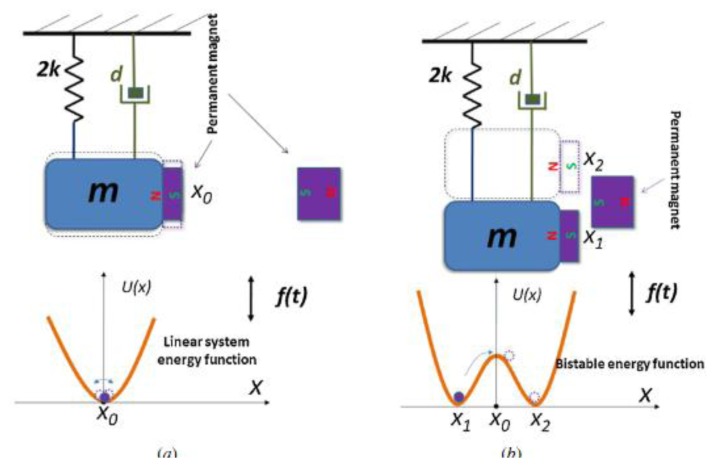
Comparison between linear and nonlinear oscillator with respect to potential energy function [96] (**^©^** IOP Publishing. Reproduced by permission of IOP Publishing).

The potential energy function *U*(*z*) can be chosen from a variety of equations [102,103]. Here, for simplicity, we report a standard quadratic equation:

(16) $$U(z)=-\alpha z^{2}+\beta z^{4}$$

where *α* and *β* are two parameters that allow determining the potential shape. The bistable behavior is described by the potential energy Function (14), reported in the lower part of Figure 23b, which shows two wells representing the stable states.

**Figure 24.**
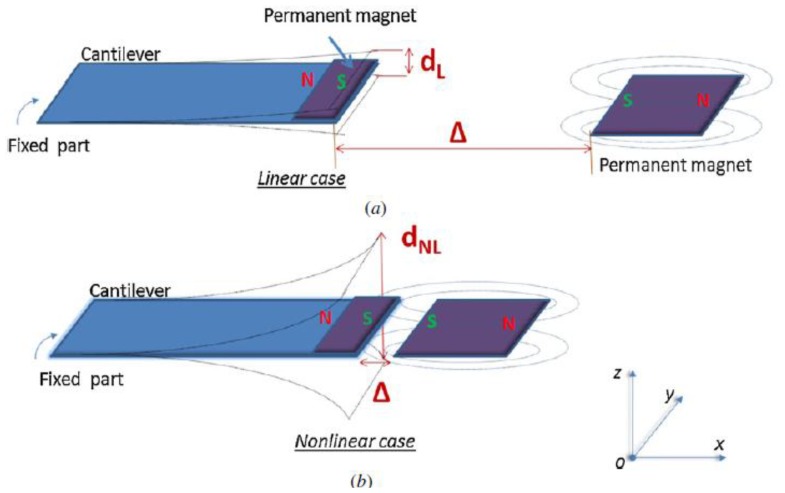
Nonlinear cantilever [96] (**^©^** IOP Publishing. Reproduced by permission of IOP Publishing).

Calling Δ the distance between magnets (Figure 24), three cases can be described:

- Large value of Δ: magnetic force is negligible and the cantilever behavior is linear, such as resonant devices. These refer to the model reported in Figure 23a;
- Small value of Δ: the external magnet is close to the cantilever tip, which is confined in one of the two stable states along the *z* axis of Figure 24. The system cannot switch between the two stable states due to the high commutation potential *U*_0_. In this case, the oscillation is small and linear around one of the two stable states.
- Medium value of Δ: in a particular range of Δ (depending on the vibration input) the system is able to commute between two stable states, which results in non-linear behavior of the device.

**Figure 25.**
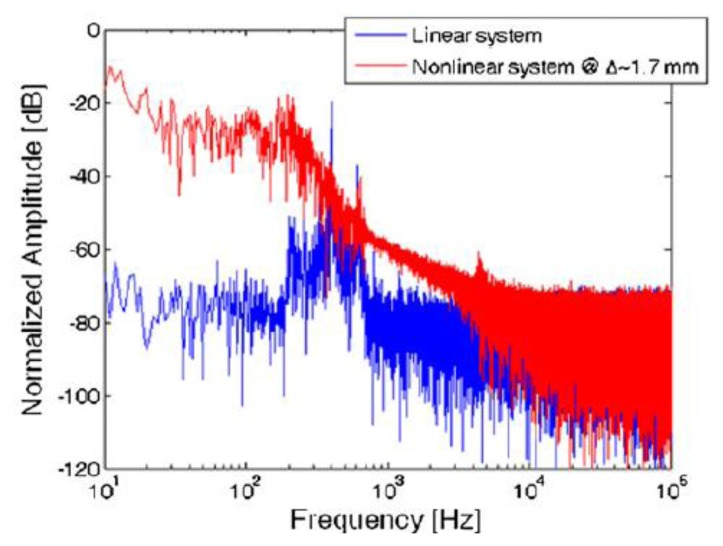
Displacement spectrum with σ = 20 μN [96] (**^©^** IOP Publishing. Reproduced by permission of IOP Publishing).

The fabricated MEMS device operated in the latter condition (nonlinear oscillation). Experimental tests resulted in the displacement spectrum (for the tip) shown in Figure 25, under an excitation input of Gaussian white noise with a standard deviation *σ* = 20 μN (that corresponds to an acceleration of 7.7 ms**^−2^**), and a distance Δ ∼1.7 mm between the magnets.

The bi-stable behavior was also achieved by involving two magnets with the same polarity of the cantilever magnetic tip [104,105]. These magnets are deployed not just in front of the tip, but shifted upwards and downwards within a frontal stage, to obtain an attraction between the magnetic tip and the two fixed magnets. Other research papers showed similar bistable systems where the switching between stable states is driven by internal signals [106,107].

Qiu and colleagues [108] demonstrated a bi-stable device consisting of a clamped-clamped buckled beam. Xu and colleagues [109] proposed a simply supported buckled beam, stating that this mechanism enhances the clamped-clamped transduction mechanism. Other bistable solutions based on buckled beam configuration were demonstrated by Arrieta and colleagues in [110] and by Cottone and colleagues in [111]. Buckled beams do not require permanent magnets and are therefore better suited for miniaturization. Andò and colleagues investigated new solutions to design bistable vibration harvesters in [112,113].

**Figure 26.**
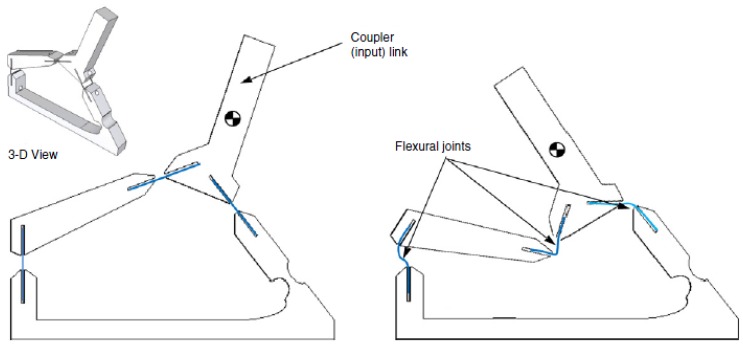
Bio-inspired bi-stable structure [114] (**^©^** IOP Publishing. Reproduced by permission of IOP Publishing).

**Figure 27.**
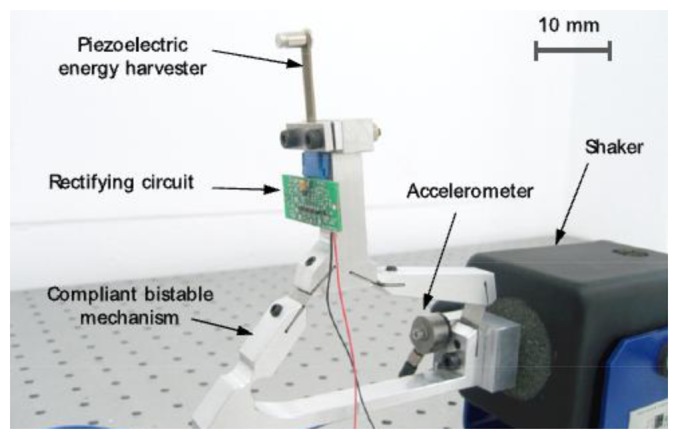
Experimental setup of the bio-inspired harvester [114] (**^©^** IOP Publishing. Reproduced by permission of IOP Publishing).

A bio-inspired device was proposed as well [114] (Figures 26 and 27). It is designed to mimic the auditory hair bundle structure. The auditory structure is responsible to stimulate the brain with electrical signals in response to the oscillations due to pressure forces of propagated sound. It mechanically amplifies the movements of the hair cells, thanks to a negative stiffness, which conveys a bi-stable behavior. The proposed device is composed by a four bar structure, linked by thin spring steel flexural joints. A piezoelectric cantilever can be placed on the coupler link.

Real harvesting applications are characterized by wide spectrum vibrations [115], with predominance of low frequencies in the case of wearable body harvesters. Non-resonant solutions allow enlarging devices bandwidth and therefore obtain effective harvesting performances, without augmenting devices area or adding power demanding self-tuning features. Moreover, bistable systems show a low-pass behavior that allows harvesting low frequency vibrations without the need for large masses and devices (necessary in resonant cantilevers). Table 3 summarizes the reviewed non-resonant devices and compares their main characteristics.

## 8. Rotational Devices

Although the piezoelectric effect is inherently related to axial elongations, several device configurations were proposed as rotatory harvesters [85,116–125], mainly by transforming a rotatory excitation into a longitudinal strain. Among these, Gu and Livermore [85] presented a compact self-tuning rotatory harvester, consisting in a rigid piezoelectric beam and a flexible beam with a tip mass at its end (a steel ball). During operation, the steel ball impacts the generating beam, letting it to vibrate. The system natural frequency is determined by the flexible beam natural frequency, which varies with the imposed centrifugal force.

**Figure 28.**
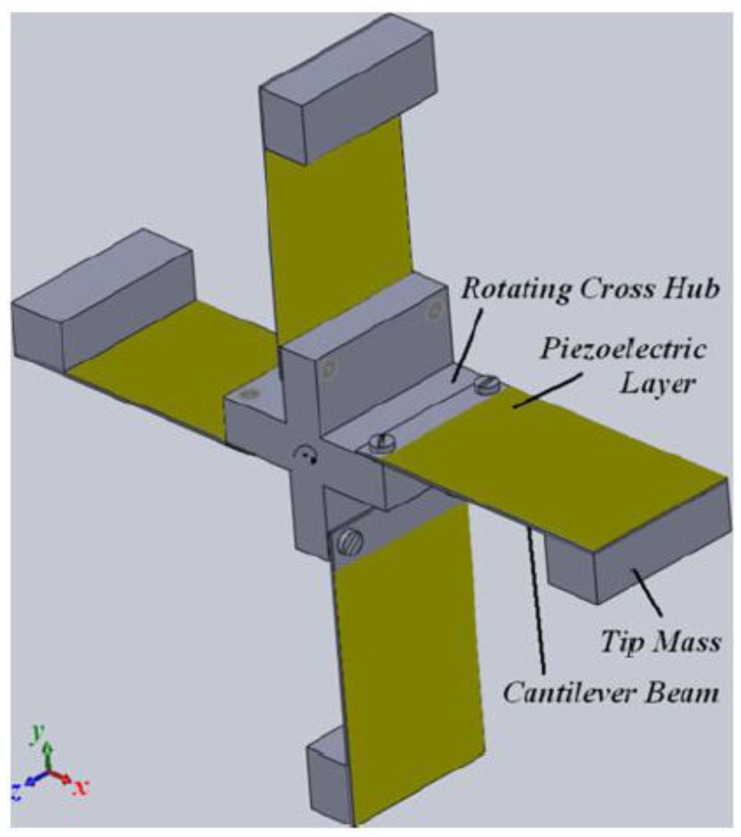
Schematic view of rotatory motion harvester (^©^ 2013 IEEE. Reprinted with permission from [120]).

Khameneifar and colleagues [119,120] applied the piezoelectric cantilever concept to a rotating shaft, as shown in Figure 28. This approach allows employing a piezoelectric device for monitoring rotating machines such as turbines or tires. The device (employing a single PZT element with a single 5 cm length cantilever beam and a 105 g tip mass) driven at 138 rad/s was able to harvest 6.4 mW on an optimum load resistance of 40 kΩ, with a corresponding output voltage of about 16 V.

Pillatsch and colleagues [121] developed a harvester composed of a fixed piezoelectric beam and an eccentric proof mass free to rotate, similar to the Seiko Kinetik watch. This device was designed for human body applications, and was tested on an upper limb during a running task. It was able to harvest up to 43 μW at 2 Hz and 20 ms^−2^.

Karami and colleagues [122] proposed two configurations of wind turbine, both consisting of a circular array of cantilever beams clamped below a windmill. These beams vibrate in response to the movement of the windmill, which is equipped with repulsive magnets.

## 9. Conditioning Circuitry

**Figure 29.**
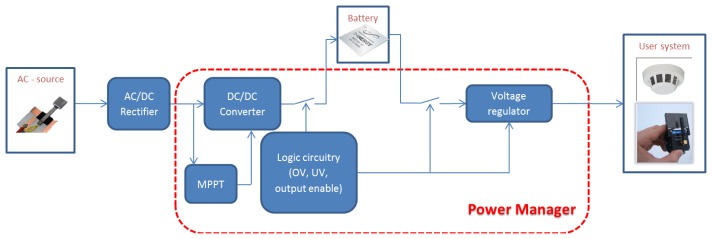
Typical schematic diagram of a power manager.

The scientific and technological challenges of energy harvesting systems also deal with power managers. These devices are responsible for transferring harvested energy from generator to a host device and in most cases to a storage element as well. A typical schematic diagram of a power manager is shown in Figure 29.

**Figure 30.**
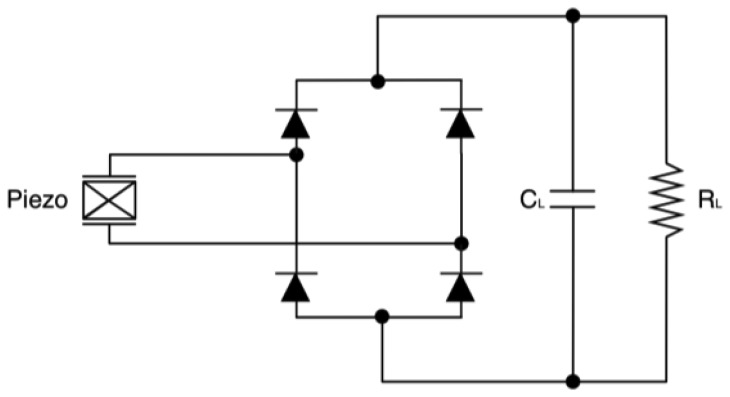
Diode bridge rectifier.

Power managers are designed both for DC and AC sources. They typically employ a DC-DC converter, mainly for impedance matching and also to match the source voltage with the battery charging level. In case of alternate sources, a rectifier is required. In case of direct current sources, impedance matching can be achieved through the use of a maximum power point tracker (MPPT). It implements algorithms apt to follow the input power peaks, which is equivalent to fix the generator operating point by acting on the equivalent impedance of the downstream circuitry. Although MPPT units are suited almost uniquely for DC sources, Yi and colleagues [126] discussed a strategy to implement an energy adaptive algorithm for vibration harvesters. The logic circuitry is designated to control and manage the charge and discharge phases of the battery. Overvoltage and undervoltage boundaries can be controlled with the help of comparators and switches. Voltage thresholds are often configurable by users using resistive dividers. Piezoelectric generators are AC sources, hence their output has to be rectified and regulated to supply host devices. The simplest rectifier can be a diode bridge rectifier (Figure 30). The AC-DC converter can be followed by a DC-DC converter, for power optimization and voltage regulation [127,128].

**Figure 31.**
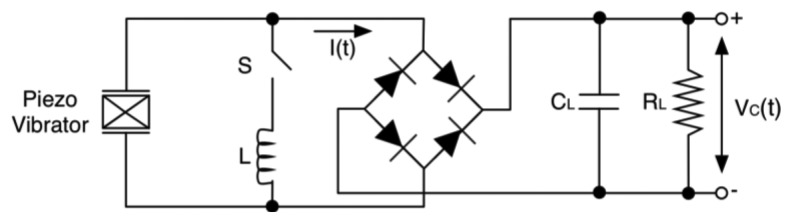
Parallel-SSHI interface.

Another possible circuital interface is the parallel-SSHI (synchronized switch harvesting on inductor) [129–131]. This approach allows to enhance the coupling coefficient of the electromechanical system using piezomaterials [130,132–134], it allows to gain up to 10 times in terms of harvested energy [135]. The technique was derived from a semi-passive technique developed for mechanical structures, called SSD (synchronized switch damping) [136–140]. Such configuration adds an inductor-switch branch in parallel to the source (Figure 31).

When the device displacement is maximum, the switch is turned ON. In this condition the internal capacitance and the inductor constitute an oscillator, where the characteristic electrical period must be chosen much smaller than the mechanical vibration period. The circuit allows to invert quasi-instantaneously the voltage of the piezoelectric element and thus to put in phase the vibration velocity and the generated voltage [141–143].

A possible implementation of the parallel-SSHI interface consists of two switches, one for the positive half-wave and another one for the negative. The switches are implemented through two MOS transistors driven by the output of a comparator that reads the derivative of the piezoelectric voltage, in order to catch the peak and let the inductor to discharge the parasitic capacitance [131].

This technique allows to dramatically increase the voltage output or to obtain the same output of a standard interface device while reducing the volume of the piezoelectric element.

**Figure 32.**
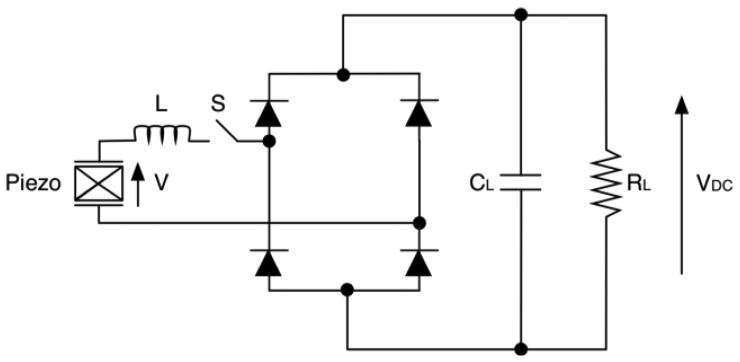
Series-SSHI configuration.

Based on the same concept, a series-SSHI rectifier consists of an inductor-switch branch added in series to the piezoelectric element, followed by a diode bridge rectifier (Figure 32). The switch control is the same as described above for parallel-SSHI.

**Figure 33.**
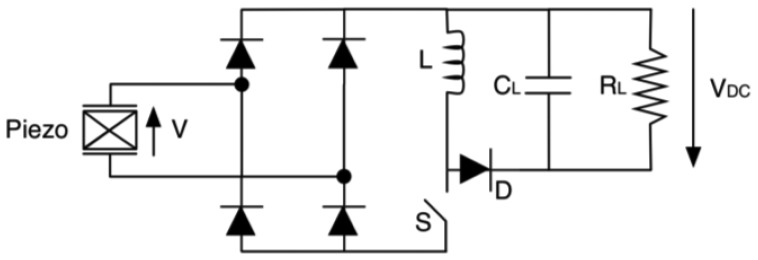
Synchronous charge extraction interface.

Lefeuvre and colleagues [28] as well reported a synchronous charge extraction interface (Figure 33). The extraction is triggered by the maxima and minima of the displacement u. When the switch is closed, the electrical energy of the internal capacitor is transferred to the inductor, and when the switch is re-opened the energy stored in the inductor is transferred to the downstream smoothing capacitor.

An experimental comparison between these configurations for a constant force amplitude input was presented in [28]. The four techniques have the same maximum harvested power, but at different values of the electromechanical coupling factor. Practically, the synchronous charge extraction technique reaches the maximum at lower electromechanical coupling factors, enabling reduction in required amount of piezoelectric materials, since *k*^2^ is roughly proportional to the amount of material. Moreover, synchronous charge extraction is indifferent to impedance matching.

**Figure 34.**
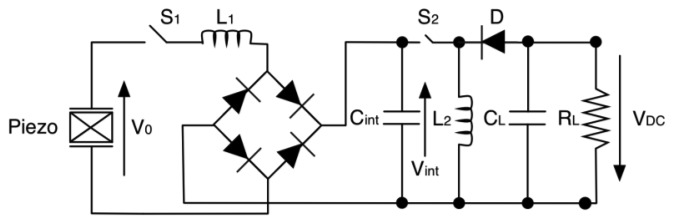
DSSH topology.

Lallart and colleagues [144] developed double synchronized switch harvesting (DSSH) by adding a buck-boost converter to the above described original idea of parallel-SSHI. This one is not driven in a conventional way. Referring to Figure 34, S_2_ is closed only when *C*_int_ is fully charged by the diode bridge rectifier (after every cycle), while S_1_ is ON when energy on L_2_ is maximum.

Several other switching techniques were reported by Guyomar and colleagues [142]. All these strategies, however, rely on a deterministic knowledge of the source frequency. If this frequency is time variant or random, the above discussed systems fail.

Giusa and colleagues [145] recently demonstrated a novel approach (called Random mechanical switching harvesting on inductor—RMSHI), which does not require deterministic synchronization. It employs a mechanical switch consisting of two mechanical stoppers. These close the circuit when the beam reaches maximum deflection. Furthermore, the mechanical switch prevents from extra voltage across the inductor, which would occur using an electronic switch.

Commercial solutions are also made available as a result of the research progresses achieved in the last years. Linear Technology (Milpitas, CA, USA) has come up with a series of conditioning devices for energy harvesting. Those circuits are targeted for piezoelectric-based harvesters [146–148] and equipped with low-loss full-wave bridge rectifier followed by a buck or a buck-boost converter. They are also provided with embedded LDOs enabling selectable output voltages, which may also be used to charge batteries.

## 10. Conclusions

Though piezoelectric energy harvesting has been thoroughly investigated since the late 1990s [149], it still remains an emerging technology and critical area of interest. Energy harvesting application fields so far mainly focused on low power devices due to their limited transduction efficiencies [150].

To date, researchers are following distinct ways in developing piezoelectric energy harvesting technology. New materials, configuration approaches and operating modes are under study, and some of these valuable solutions were proposed in order to achieve large bandwidth harvesters that are able to scavenge energy from diverse environments.

Resonant *d*_33_ cantilever beams need optimization, but several interesting solutions and approaches that were published can push forward the research. *d*_15_ harvesters are still too complex to be fabricated, but exhibit great potential.

In this review paper these configurations have been briefly studied using a comparison table with respect to *d*_31_, reporting various factors. From this analysis we can conclude that *d*_15_ is more efficient than other modes, *d*_33_ has higher output voltages, simplifying the power conversion process, whereas *d*_31_ is the simplest solution found in terms of fabrication process and performances (in most cases).

Considering nanoscale harvesters, they represent a promising but still emerging technique that requires to be consolidated. Non-resonant solutions, as well as frequency tuning methods, are powerful instruments to push forward the growth of vibration harvesting techniques. However, though several non-resonant solutions were demonstrated, new roads can be explored. As an example, in electromagnetic vibration harvesting a well-known technique to achieve bistability involves mechanical bumpers [151,152]. Furthermore, all these piezoelectric harvesting research branches could be merged. Likely, a bistable harvester involving a high efficiency *d*_15_ material, equipped with a proper conditioning circuitry, would achieve significant results.

The limit in terms of harvested energy density has still to be overcome. This has been the main technological challenge so far. A well-integrated roadmap was designed in the framework of the Guardian Angels Coordination Action within the Future and Emerging Technologies Flagship initiative funded by the European Commission [153]. In this framework, research efforts are focusing on the transducer and also on the integration with the downstream conditioning circuitry, power management circuits and application devices.

## Figures and Tables

**Table 1.** Coefficients for common piezoelectric materials [21,37–42]. Ceramic B is a modified barium titanate with improved stability and lower aging. Last two rows report strongly different values for two PMN-PT crystals; notice that this is not due to the slightly different stoichiometric composition but to the crystallographic cut (not reported in the table).

**Compound**	***d****_33_*	***d****_31_*	***d****_15_*	***g****_33_*	***g****_31_*	***g****_15_*	**Curie Point [°C]**

	**[10^−12^ C N^−1^]**			**[10^−3^ V m N^−1^]**			
PZT-2	152	−60.2	440	38.1	−15.1	50.3	370
PZT-4	289	−123	496	26.1	−11.1	39.4	328
PZT-5A	374	−171	584	24.8	−11.4	38.2	365
PZT-5H	593	−274	741	19.7	−9.1	26.8	193
PZT-8	225	−37	330	25.4	−10.9	28.9	300
Pz21	640	−259	616	15.6	−7.4	26.8	218
Pz23	328	−128	421	24.7	−9.6	34.3	350
Pz24	149	−58	247	39.7	−15.4	37.7	330
Pz26	328	−128	327	28	−10.9	38.9	330
Pz27	425	−170	506	26.7	−10.7	37.3	350
Pz28	275	−114	403	31.4	−13	37.3	330
Pz29	574	−243	724	22.6	−9.6	32.1	235
Pz34	46	−5.33	43.3	25	−2.9	27.9	400
Ceramic B	149	−58	242	14.1	−5.5	21	115
BaTiO_3_	145	−58	245	13.1	−5.2	20.5	120
PVDF	−33	23	-	330	216	-	100
0.70PMN-0.30PT	1,611	−2,517	157	29.2	−45.6	9	150
0.69PMN-0.31PT	-	-	5,980	-	-	56	146
MFC M8528	460	−210	-	-	-	-	80

**Table 2.** Resonant devices comparison. The last column reports the power factor, *i.e.*, the output power density normalized with the input acceleration (expressed in *g*=9.81 *m/s*^2^).

**Reference**	**Material**		**d_ij_ [pm/V]**	**V_device_ [mm^3^]**	**V_piezo_ [mm^3^]**	**f_n_ [Hz]**	**R_L_ [Ω ]**	**V_out_ [V]**	**P_density_ [W/cm^3^]**	**Power Factor [W/(g^2^cm^3^)]**

	**Name**	**Mode**								
[78]	AlN	d31	-	0.5	0.0004	1.4 × 10^3^	650 × 10^3^	1.6	4 × 10-^3^	248 × 10-^6^
[48]	PZT	d31	320	212.5	80.4	223.8	9.9 × 10^3^	-	77 × 10^-6^	1.4 × 10-^6^
[61]	PZT	d33	-	-	0.00002	13.9 × 10^3^	5.2 × 10^6^	2.4 dc	-	-
[63]	PZT	d33	100	3.5	0.014	118.1	4.5 × 10^6^	4.7	136 × 10^-6^	543 × 10-^6^
	PZT	d31	-55	3.5	0.014	130.8	11 × 10^3^	0.77	1.9 × 10-^3^	7.8 × 10-^3^
[57]	PZT	d33	100	1.1	0.003	243	2 × 10^6^	2 rms	1.6 × 10^-3^	6.4 × 10^-3^
	PZT	d31	-55	1.1	0.003	243	9.9 × 10^3^	1.5 rms	2 × 10-^3^	8.1 × 10-^3^
[77]	PZT	d15	700	171.2	65	73	2.2 × 10^6^	6.2	51 × 10-^6^	-
[76]	PZT	d15	741	4.8	2.5	45	1.6 × 10^6^	19 × 10^-3^ rms	87 × 10-^6^	67.8 × 10-^3^
[74]	PMN-PT	d15	3,080	24,100	156	-	91 × 10^3^	11.3	29 × 10-^6^	189 × 10^-9^
[35]	ZnO	d31	10	-	9.7	10 × 10^6^	500 × 10^6^	8 × 10-^3^	4 × 10-^6^	624 × 10-^15^
[68]	MFC	d33	400	340	120	22.5	4 × 10^6^	-	494 × 10-^6^	17.1 × 10^-3^
[69]	PVDF	d33	33	549.5	24.2	1.5	10 × 10^6^	-	364 × 10-^6^	-

**Table 3.** Comparison of some non-resonant devices. Bistable devices can be benchmarked based on the bistability mechanism. Magnetic repulsion/attraction devices show large bandwidths, but the use of magnets limits technological scaling (magnetic attraction devices require double number of magnets than magnetic repulsion-based harvesters). Buckled beam-based harvesters employ a snap-through mechanism, and are better suited for integration. The main drawback of bistable devices is that they require a particular amount of energy to overcome the potential barrier and switch between the two stable states.

**Reference**	**Bistability Mechanism**	**Advantages**	**Drawbacks**
[96]	Magnetic repulsion	Large bandwidth at low frequencies (0∼100 Hz); MEMS	Input force threshold to achieve bistability; lower response than resonant device at its natural frequency
[105]	Magnetic attraction	High power output at less than 10 Hz	Input force threshold to achieve bistability; lower response than resonant device at its natural frequency; number of magnets
[108]	Clamped-Clamped buckled beam	No hinges, internal stress or magnets required; MEMS	Input force has to exceed the buckling load
[109]	Simply supported buckled beam	No hinges, internal stress or magnets required; improved transduction mechanism	Input force has to exceed the buckling load
[114]	Bio-inspired by auditory system	Snap-through mechanism, independent from excitation frequency; well suited for 1∼10 Hz harvesting	Advanced mechanical structure with a commercial piezoelectric harvester
